# Cerebello-thalamo-cortical network is intrinsically altered in essential tremor: evidence from a resting state functional MRI study

**DOI:** 10.1038/s41598-020-73714-9

**Published:** 2020-10-07

**Authors:** Valentina Nicoletti, Paolo Cecchi, Ilaria Pesaresi, Daniela Frosini, Mirco Cosottini, Roberto Ceravolo

**Affiliations:** 1 Department of Clinical and Experimental Medicine, University of Pisa, via Roma 67, 56126 Pisa, Italy; 2 Neuroradiology Unit, Pisa University Hospital, Pisa, Italy; 3 Department of Translational Research and New Technologies in Medicine and Surgery, University of Pisa, Pisa, Italy

**Keywords:** Diseases, Neurology

## Abstract

Cerebello-thalamo-cortical network is suggested to be involved in the pathophysiology of Essential Tremor (ET). 23 patients with ET and 23 matched HC underwent a 3T-MRI with acquisition of a resting state sequence. Connectivity was investigated using a seed-based regression analyses approach. In ET patients were observed:

- **Reduced connectivity between left primary motor cortex (M1) seed and right premotor cortex and cerebellum and bilateral premotor, parietal areas, supplementary motor area (SMA);**
- I**ncreased connectivity between left somatosensory cortex (S1) seed and parietal areas, M1, premotor cortex, SMA; reduced connectivity of this seed with cerebellum.**
- **Increased connectivity of SMA seed with premotor cortex and decreased with parietal and precentral areas;**
- **Increased connectivity between left thalamus seed and cerebellum;**
- **Reduced connectivity between right cerebellum seeds and other cerebellar areas, precentral and premotor areas**.

ET showed altered connectivity within the cortical sensory-motor network and between cerebral cortex and cerebellum. The increased connectivity between cerebellum and thalamus is consistent with their crucial role in tremor generation. These findings support the dynamical entrainment of multiple central oscillators throughout the cerebello-thalamo-cortical network in ET. This evidence is strengthened by the finding that this network is altered also when the core symptom is absent.

## Introduction

Essential Tremor (ET) is one of the most common movement disorders in adults and it is characterized by postural and kinetic tremor mainly involving upper limbs. Electrophysiological^1^ and imaging^2^ studies have strongly supported the role of cerebello-thalamo-cortical network in the generation of tremor but they did not unveil the source of oscillatory activity in this disorder.

A key function has been proposed for cerebellum considering that ET patients frequently show clinical features suggestive of cerebellar involvement such as ataxic gait^3^, intention tremor^4^, eye movement abnormalities^5^. Furthermore, although pathological and morphological imaging studies provided controversial results as regards structural changes of cerebellum^6,7^, cerebellar functional abnormalities have been clearly revealed by both PET^8^ and functional MRI (fMRI) works^2,9^.

Nonetheless the efficacy of thalamotomy and deep brain stimulation (DBS) of ventral intermediate nucleus (VIM) of thalamus in the treatment of drug-resistant ET, have focused attention on this structure as a possible key element in tremor generation^10,11^. Some electrophysiological studies supported this hypothesis by unveiling a neuronal discharge activity in VIM synchronous to EMG activity registered from opposite arm^12,13^. Additionally, evidences of a cerebral cortex involvement in pathogenesis of ET have been provided. Several high-resolution electroencephalography (EEG) and magnetoencephalography (MEG) studies showed that cortical activity is coherent with ET^1,14^ and subdural stimulation of motor cortex alleviates tremor in ET patients^15^.

Thus, all nodes of cerebello-thalamo-cortical network seem to be similarly involved in tremor oscillations and evidences of alleviation of tremor after stroke anywhere in the circuit prompted the hypothesis of a mutual entrainment between network components. Invasive tract-tracing studies in normal non-human primates^16^ and non-invasive diffusion tensor imaging (DTI) evidences in healthy humans^17^ have demonstrated the existence of an anatomical network which connects cerebral motor cortex, VIM and cerebellum. Further a functional network similar to this anatomical circuit has been described in healthy humans by using functional connectivity (FC) analysis of resting-state fMRI (rs-fMRI)^18,19^. Rs-fMRI overcomes limitations of task-based fMRI studies by allowing the investigation of large scale functional networks based on the temporal correlations of spontaneous, no task related, blood oxygen level dependent (BOLD) fluctuations in a very low frequency range. The analysis of FC in rs-fMRI allows also to avoid the potential noise in BOLD signal fluctuations originating from sensorial input and motor output linked to the execution of a motor tasks and the presence of tremor and it is helpful to study movement disorders.

We performed a rs-fMRI study comparing ET patients and healthy controls (HC) to investigate the different components of the network involved in the generation of the oscillatory activity and thus to explore the hypothesis that a network, rather than a single structure, is the tremor generator of ET.

## Methods

### Participants

Twenty-three ET patients [13 males and 10 females; 71.6 ± 10.5 years] were recruited at the Movement Disorders Center of our University. All patients had a diagnosis of definite or probable ET according to Consensus Statement on tremor of Movement Disorder Society^20^. Exclusion criteria were thyroid dysfunctions, severe vascular encephalopathy, recent brain injuries, structural lesions potentially related to tremor or history of tremorogenic drugs use, psychogenic tremor, dystonic features, dementia. Twenty-three healthy volunteers (11 males and 12 females; 70.3 ± 5.3 years) with no history of neurological or psychiatric diseases were recruited as HC.

All patients and HC were right-handed according to Edinburgh Handedness Inventory and gave their written informed consent to the study protocol. It was approved according to the rules of the local Ethics Committee, which name is *Comitato Etico Area Vasta Nord Ovest—Azienda Ospedaliero Universitaria Pisana*. The experiments were carried out according to the guidelines and regulations.

### Clinical assessment

Each patient was evaluated by a neurologist expert in movement disorder (VN, 8 years of experience) on the same day of the MRI examination.

Clinical evaluation included the Fahn-Tolosa-Marin rating scale (TRS) and Mini Mental State Examination (MMSE). Medications for tremor were stopped 7 days before the MRI scan, thus, all patients were clinically scored in the same condition (off medication). MMSE evaluation was also performed in HC. Demographic characteristics of patients and HC are reported in Table 1.

**Table 1:** Demographic and clinical features of ET patients and HC.

	ET patients	HC
Number #	23	23
Age (years)^a^	71.6 ± 10.5	70.3 ± 5.3
Gender	13 males; 10 females	11 males; 12 females
Disease duration (years)^a^	14.9 ± 13.4	–
Age of onset (years)^a^	56.7 ± 17.1	–
MMSE^a^	28.0 ± 1.6	28.6 ± 0.9
**Body parts with tremor**		
Upper limb #	23	–
Lower limb #	7	–
Head #	9	–
Voice #	8	–
TRS (A + B)^a^	22.3 ± 8.9	–
Patients with family history #	12	–
Patients on therapy #	10	–
Type of therapy	7 Propranolol 2 Gabapentin 1 Zonisamide	–

^a^Expressed as mean ± standard deviation.

### Data acquisition

Images were acquired on a 3T scanner (Discovery MR750 3T, GE Healthcare, Milwaukee) equipped with an 8-channels head coil with ASSET technology. Rs-fMRI data were acquired using a T2* weighted gradient recalled echo-planar imaging (EPI) sequence (TR 2100 ms, TE 40 ms, flip angle 90°, FOV 260 mm, matrix size 128 × 128) with 28 interleaved slices (thickness 4 mm, spacing 1 mm) angled of 30° with respect to the anterior–posterior commissural plane (AC-PC) to minimize susceptibility artefacts, repeated over 180 volumes (total scanning time of 6′ 18″). A high-resolution 3D Spoiled Gradient Recalled sequence was also acquired on axial plane (TR 8.2 ms; TE 3.2 ms; flip angle 12°; TI 450 ms; FOV 256 mm; matrix size 256 × 256; 160 slices; thickness 1 mm). We asked subjects to maintain their eyes closed and to remain awake.

### Seed-based functional connectivity (FC) analysis

Rs-fMRI data were processed using FEAT tool part of FSL package. After discarding the first 4 volumes, each dataset underwent preliminary processing including removal of non-brain structures (by using BET^21^), slice-timing correction, motion correction (by using MCFLIRT^22^), spatial smoothing (Gaussian kernel of Full Width Half Maximum = 8 mm), high pass temporal filtering (cut off = 100.0 s). Subjects with absolute translational or rotational displacement higher than 3 mm or 3° were excluded from further analysis. FC was investigated with seed-based regression analysis. Seeds were selected according to a recent motor task-driven fMRI study performed in ET patients and HC^2^ which showed statistically significant differences in left precentral gyrus (M1), supplementary motor area (SMA), postcentral gyrus (S1), posterior thalamus and right cerebellum (lobule IV–V; lobule VI, lobule VIII). The selected ROIs were only those which significantly differed in ET patients without resting tremor compared to healthy controls. In these areas 7 spherical ROIs were drawn around the voxel with maximum value in the Z statistical maps resulting from the between-groups analysis. All seeds were created in the Montreal Neurological Institute (MNI) 152 standard space and co-registered to the functional dataset of each subject by affine transformations (FLIRT^23^) in order to extract, for each subject, the mean timeseries for each seed.

Structural images were brain-extracted using BET^21^ and segmented into grey matter (GM), white matter (WM) and cerebrospinal fluid (CSF) by FAST tool^24^. WM and CSF images were thresholded at 80% tissue type probability in order to create WM and CSF masks that were co-registered to functional images by affine transformation in order to extract the mean timecourses for WM and CSF averaging the time series of all voxels within the masks. These time series were used as regressors in the statistical model to remove the corresponding artifactual signals from the analysis.

For the FC analysis we carried out a 7 multiple regression analysis (using General Linear Model implemented in FEAT) with seed timecourse regressors and 8 nuisance covariates (6 motion parameters, CSF signal and WM signal) for each subject. Positive statistical correlation map was obtained for each seed and registered by affine transformation to MNI template. Then within-group and between-group analysis were performed by a Fixed Effect model approach^25^. Corrections for multiple comparisons were applied at the cluster level by using Gaussian random field theory. We also accounted for multiple seeds by Bonferroni correction method (min Z > 3.3; cluster significance: *p* < 0.05/7 = 0.007). The results were adjusted for age and sex. In ET patients’ group we also looked for relationships between FC strength and clinical variables such as disease duration and tremor severity assessed by TRS (part A + part B). We implemented one sample t-test by Fixed Model in FSL. The resulted thresholded Z statistical maps were corrected at the cluster level using Gaussian random field theory and Bonferroni correction (Z > 3.3, *p* < 0.007).

## Results

All participants showed absolute translational or rotational displacement lower than 3 mm or 3° and the mean absolute head movement was below 1.5 mm, so all the participants were evaluated in the final analysis. ET patients and HC groups did not significantly differ in age (Mann–Whitney U test *p* = 0.91), gender (Test di Fisher *p* = 0.77) and MMSE score (Mann–Whitney U test *p* = 0.23). Any ET patient exhibited resting tremor.

### Seed-based FC analysis: between-groups

**Figure 1:**
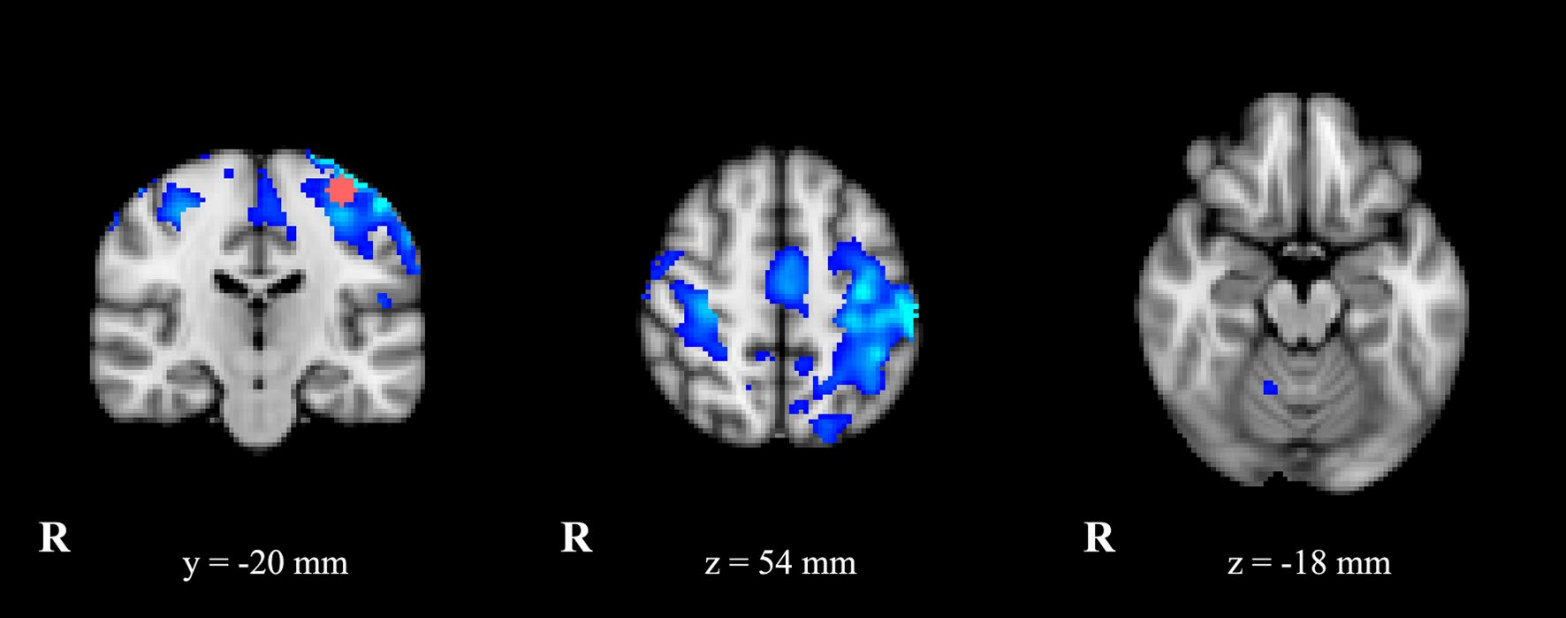
Statistical comparison maps between ET patients and HC for left M1-seed (ROI seed in pink). FC analysis showed a reduced connectivity (in blue) of left M1 with premotor cortex, SMA, somatosensory areas and cerebellum in patients compared to controls. Results are superimposed on axial and coronal slices of standard MNI template (Z threshold > 3.3, cluster *p* significance < 0.007).

**Figure 2:**
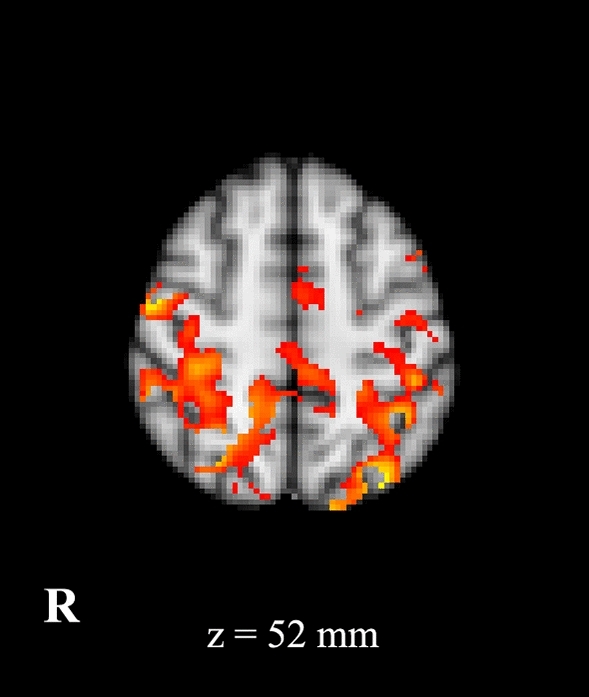
Statistical comparison maps between ET patients and HC for left S1-seed. An increased connectivity (in red) between somato-sensory cortex and parietal areas, primary motor cortex and SMA was observed in patients as compared to controls. Results are superimposed on axial slice of standard MNI template (Z threshold > 3.3, cluster *p* significance < 0.007).

- *Left M1 seed* showed in ET patients with respect to HC reduced FC with right precentral gyrus and with bilateral superior and middle frontal gyri, SMA, middle cinguli gyrus, postcentral gyrus, superior and inferior parietal gyri and supramarginal gyrus. When bilateral, the reduction of connectivity was greater on the left side. Beside M1 showed in ET patients bilateral reduced connectivity with precuneus and paracentral lobe without any side-predominance. Finally, M1 exhibited in ET reduced FC with right cerebellum (lobules IV–V and VI) (Fig. 1, Supplementary Table 1).
- *Left S1 seed* exhibited in ET patients compared to HC increased connectivity bilaterally with precentral gyrus, superior and middle frontal gyri, Rolandic operculum, SMA, middle cinguli gyrus, superior and inferior parietal gyri, supramarginal, angular gyri, precuneus and paracentral lobule. It showed also increased FC with right postcentral gyrus in ET (Fig. 2). Further left S1 seed displayed in ET patients compared to HC decreased FC with cerebellum, specifically with right lobules IV–V, VIII and lobule VI bilaterally (Supplementary Table 2).
- *Left SMA seed* demonstrated less FC with bilateral precentral gyrus, postcentral gyrus, superior and inferior parietal gyri and supramarginal gyrus in ET patients with respect to HC. On the other side SMA seed showed increased FC with superior, middle and inferior frontal gyri, medial superior frontal gyrus and cinguli gyrus bilaterally and with right SMA in ET patients compared to HC (Supplementary Table 3).
- *Left thalamus seed* showed increased FC with cerebellum, particularly with lobule VI bilaterally, in ET with respect to HC (Supplementary Table 4).

Within the cerebellum we defined three seed regions: right lobule IV–V, lobule VI and lobule VIII according to our previous findings^7^.

**Figure 3:**
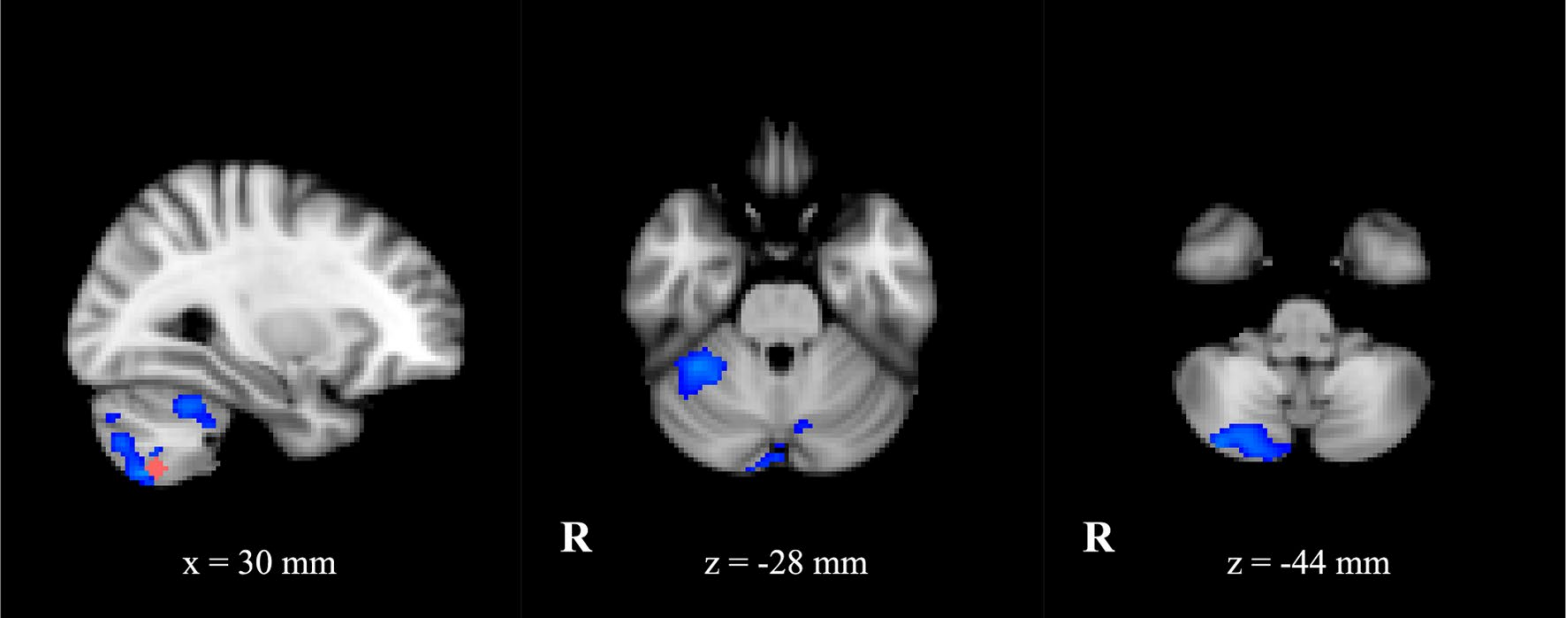
Statistical comparison maps between ET patients and HC for lobule VIII-seed (ROI seed in pink). FC analysis showed a decreased connectivity (in blue) between cerebellar hemispheres each other in patients compared to controls. Results are superimposed on axial and sagittal slices of standard MNI template (Z threshold > 3.3, cluster p significance < 0.007).

- *Lobule IV–V seed* in ET patients demonstrated reduced FC compared to HC with precentral gyrus, Rolandic operculum and SMA bilaterally and with left superior frontal gyrus. It showed also reduced FC with other cerebellar areas: crus 1, crus 2, lobules VI, VIIb, VIII, IX and X bilaterally (Supplementary Table 5).
- Likewise, *lobule VI seed* exhibited reduced FC in ET patients compared to HC with other regions of cerebellum: crus 1 bilaterally (more widespread on the right side), right crus 2, lobules IV–V, VIIb, VIII, IX and X. This seed showed reduced FC also with cortical areas such as left precentral gyrus and superior frontal gyrus (Supplementary Table 6).
- *Lobule VIII seed* exhibited in ET patients with respect to HC reduced FC with other regions of cerebellum: crus 1 and crus 2 bilaterally, right lobules VI and VIIb (Fig. 3, Supplementary Table 7).

### Relationship of functional connectivity and clinical variables in ET patients

The FC strength of *M1 seed* showed a negative relationship with tremor severity in bilateral premotor areas, in bilateral SMA, right thalamus and cerebellum (bilateral crus 1 and lobule VI; left crus 2). Furthermore, FC of *M1 seed* increased parallel with tremor severity in bilateral posterior parietal areas (Supplementary Table 8a). The FC strength of *M1 seed* showed a negative relationship with disease duration in bilateral premotor areas and right SMA (Supplementary Table 8b).

With regard to *thalamus seed*, its FC with bilateral cerebellum had a positive relationship with tremor severity and disease duration whereas its FC with bilateral fronto-parietal areas showed a negative relationship with TRS (Supplementary Table 9).

The FC strength of *SMA seed* with precentral gyrus negatively correlated with TRS and disease duration (Supplementary Table 10).

The FC of cerebellar *lobule IV–V seed* with other cerebellar areas, left M1, bilateral premotor areas and right SMA showed a negative relationship with tremor severity (Supplementary Table 11a). Further the FC of this seed region with left M1 and other cerebellar areas correlated negatively with disease duration (Supplementary Table 11b). The FC strength of cerebellar *lobule VI seed* as well as of cerebellar *lobule VIII seed* showed a negative relationship with tremor severity in other cerebellar areas, left M1 and premotor areas and a negative relationship with disease duration in other cerebellar areas (Supplementary Tables 12, 13).

## Discussion

The results of our study strongly support the hypothesis of a mutual entrainment between the components of cerebello-thalamo-cortical circuit in generating tremor in ET. More specifically, a physiological network turned out to be basically altered in ET patients with respect to HC. Several studies showed functional activity changes in cerebello-thalamo-cortical circuit during the execution of motor tasks in ET patients compared to HC^2,26,27^. However, we demonstrated that this network is basically perturbed in ET patients, also in rest conditions when tremor is absent. Furthermore, in our work all nodes of the circuit showed aberrant functional connections contributing to the network dysfunction. This result, although reported in a limited sample of ET patients, could be unsupportive for the existence of a unique central oscillator dysfunction. The hypothesis of the involvement of multiple generators pertained to the cerebello-thalamo-cortical network is also supported by PET and fMRI studies^10,28,29^ and mathematical models^30,31^ which investigated therapeutic effects of DBS of VIM thalamic nucleus in ET patients.

Our study showed a reduced FC between primary motor cortex (M1) and contralateral cerebellum, lobule IV–V and VI, which correspond to the motor part of cerebellum. This data is in line with two motor-task driven fMRI studies where decreased FC between cortical motor areas and motor cerebellum in ET patients compared to HC were demonstrated^26,27^. Our finding of a decreased functional coupling between motor cortex and cerebellar motor areas also in rest condition, gives further strength to its role in tremor generation in ET. Furthermore, as previously found^26^, FC between M1 and motor cerebellum correlated negatively with tremor severity.

The decreased FC between cortical motor areas and motor cerebellum is bidirectional as it is present independently from the selected seed. Lobule IV–V alone also exhibited reduced FC with SMA. It should be considered that cerebellar lobules IV and V are anatomically (and not only functionally) connected with M1^32^ and they are involved in the execution of voluntary, goal-directed movements^33^. Our finding replicates the evidence of a disrupted connectivity between cerebellum and cortex^26^ further demonstrating this alteration of connectivity also in rest conditions in ET patients. We additionally confirmed, as previously shown^26^ that tremor severity correlates with M1-cerebellar bidirectional disconnectivity. It has been suggested that a reduction of the inhibitory output of cerebello-cortical network could result in an increased cerebello-thalamic connectivity^26,27,34^. Actually cortical cerebellar GABAergic neurotrasmission dysfunction related to tremor severity has been demonstrated^35^ and reduction of Purkinje cells’ number has been shown by several pathological studies although the results are not univocally agreed^36^. Purkinje cells constitute the unique output of cerebellar cortex and lead to cerebellar deep nuclei, principally to dentate nucleus. A fMRI study performed by using finger-tap motor task showed increased dentate activity which was correlated with increasing of tremor severity^37^. Thus it could be speculated that a pathological entrainment of cerebello-thalamo cortical network could result from a disinhibited dentate nucleus^26^.

Cerebellum exhibited also an abnormal connectivity within itself: lobule IV–V, VI and VIII showed decreased FC each other and with other cerebellar areas ipsi- and contra-laterally in ET compared to HC, as already found in several previous motor-task driven fMRI studies^27,37^. The role of cerebellum as crucial point in the generation of ET is supported by several studies^35,38^ and even the positive effect of VIM-DBS on tremor has been linked to the reduction of inhibitory cerebellar connectivity^37^. However previous reports^39^ and our research cannot definitely conclude about a primary role of cerebellum in ET generation.

Our study showed a wider aberrant connectivity within the cerebello-thalamo-cortical network. Primary motor cortex exhibited a reduced FC also with premotor, parietal areas, SMA as well as SMA showed altered FC with primary motor, premotor and parietal areas. Parietal areas themselves demonstrated increased functional connectivity with primary motor, premotor and posterior parietal areas, SMA. These data could suggest pathological changes in cortical integrative processing. Cortical involvement in the pathogenesis of tremor has long been questioned. High-resolution EEG/MEG studies showed cortical activity which was coherent with tremor registered by EMG and reflected rhythmic cortical output^1,14^. In our study FC for M1-seed with SMA and premotor areas resulted decreased in ET with respect to HC with a negative correlation with tremor severity. This observation suggests a pathogenic role of cortical motor areas whose interplay is basically perturbed in ET patients. However, motor cortex could not be the unique generator of tremor as suggested by an electrophysiological study demonstrating that cortical participation in the generation of tremor is intermittent whereas characteristics of tremor did not change^14^. Our data exhibited also the involvement of secondary motor cortices as coherence between premotor activity and tremor frequency has been reported in ET patients^1^. Particularly we demonstrated a reduced FC between SMA-seed and both primary motor cortex and parietal areas whereas this seed showed increased FC with premotor areas. A reduced connectivity between SMA and M1 has recently been found in ET patients in a resting state-fMRI study^40^ as well as an abnormal SMA activation has been demonstrated in ET during the execution of motor task^27^. Taking into account that cerebellum exhibits connections with SMA, lateral premotor cortex and cingulate motor areas, SMA could contribute to the oscillatory network by transmitting abnormal cerebellar output. A compensatory role of SMA in cerebello-thalamo-cortical network has also been proposed^40^ considering the downregulated connection between SMA and M1 as a tentative response to the enhanced FC between these two regions in task-related studies^27,41^. In our work, we did not find any altered connectivity between SMA and cerebellum. Otherwise the decreased FC between SMA-seed and M1 in ET patients negatively correlated with TRS and duration of disease, supporting a participation of SMA in the oscillatory network. More recently a modulating role of SMA in the appearance of head tremor in ET patients has been proposed by a rs-fMRI study^42^.

Noteworthy cortical involvement extends beyond motor areas. Indeed our work showed the involvement of posterior parietal areas such as superior and inferior parietal gyri, supramarginal, angular gyri, precuneus and paracentral lobule. These data are consistent with a widespread functional network extending beyond the cerebello-thalamo-motor cortical pathway and associated with tremor severity in patients with ET as showed by other functional^43,44^ and morphometric^45^ MRI studies. All these findings could also suggest a deficiency in ET in integrating multi-modal information in the fronto-parietal network and thus the existence of pathological changes in cortical integrative processing. This altered cortical integrative processing suggested by the anomalous FC between primary and secondary motor cortices and somatosensory areas, could independently participate, along with cerebellum, to the aberrant oscillatory activity within cerebello-thalamo-cortical network generating ET^46^.

The widespread involvement beyond motor areas of cerebello-thalamo-cortical network could persuade to hypothesize a relationship with motor signs other than kinetic tremor and no motor symptoms, often described in ET patients^47^. However the participants of our study were all “pure ET patients” showing only kinetic tremor in upper limbs and possibly in other body’s segments without either other neurological signs nor cognitive decline (see Table 1). Thus we could speculate that changes we found in the cerebellar, thalamic and cortical areas of the network represent the neuronal correlates of the cardinal symptom of ET, kinetic tremor.

Finally, our study confirmed also thalamic role in the pathogenesis of tremor. Actually, as previously demonstrated in a motor task-driven fMRI study^18^, left thalamus-seed showed increased FC with cerebellum and the connectivity between these two structures showed a positive relationship with tremor severity. Further altered FC between VIM and cerebellum has been shown in two rs-fMRI^34,48^. VIM neurons’ discharge synchronous to tremor frequency has also been electrophysiologically registered^13^ but this fact did not necessarily mean that thalamus actively participate to the generation of tremor. VIM could simply rely sensory inputs from periphery as well as it could be driven by another generator which simultaneously influenced muscular activity. However, the efficacy of thalamotomy and DBS of VIM in improving drug-resistant tremor of ET patients, suggested that almost a subpopulation of thalamic neurons actively contribute to tremor generation. As previously highlighted, VIM-DBS produces functional changes within distal nodes of the cerebello-thalamo-cortical circuit, revealed both by PET^28,29^ and fMRI^10^ studies. The latter especially showed that long-term therapeutic effectiveness of DBS correlated with functional activation in sensorimotor cortex, SMA, thalamus, cerebellum and brainstem, all established nodes of tremor circuit, confirming the network-wide modulation effect of DBS. Recently the rise of MRI-guided high-intensity focused ultrasound (MRgFUS) thermal ablation technique to produce a thalamotomy allowed the chance to use fMRI to study the changes in the brain network caused by a focal lesion. Two rs-fMRI studies performed in drug-resistant ET patients treated with MRgFUS, confirmed connectivity changes in the motor tremor network and not only in the target region^49,50^.

In conclusion our findings support the hypothesis that multiple central oscillators throughout the cerebello-thalamo-cortical network dynamically entrain each other to generate tremor in ET. The evidence that this whole network is altered not only when investigation is performed in the presence of tremor but even when ET is investigated in the absence of the core symptom as in our study suggests that all such nodes play a primary role.

## Supplementary information

Supplementary Tables.
