# Supplementary material for: Cerebello-thalamo-cortical network is intrinsically altered in essential tremor: evidence from a resting state functional MRI study
Source: Sci Rep. 2020 Oct 7;10:16661. doi: 10.1038/s41598-020-73714-9 (PMC7541442; doi:10.1038/s41598-020-73714-9)
Supplement: Supplementary file 1 — Supplementary Tables. [file 41598_2020_73714_MOESM1_ESM.pdf]

Cerebello-thalamo-cortical network is intrinsically altered in essential tremor: evidence from a resting state functional MRI study

*Valentina Nicoletti, Paolo Cecchi, Ilaria Pesaresi, Daniela Frosini, Mirco Cosottini, Roberto Ceravolo**

**Corresponding author: Department of Clinical and Experimental Medicine, University of Pisa, via Roma 67, 56126, Pisa, Italy. E mail address: r.ceravolo@med.unipi.it*

Supplementary material:

Supplementary Table 1……………………………………………………………….2

Supplementary Table 2……………………………………………………………….3

Supplementary Table 3……………………………………………………………….5

Supplementary Table 4……………………………………………………………….6

Supplementary Table 5……………………………………………………………….7

Supplementary Table 6……………………………………………………………….8

Supplementary Table 7……………………………………………………………….9

Supplementary Table 8………………………………………………………………10

Supplementary Table 9………………………………………………………………12

Supplementary Table 10……………………………………………………………..14

Supplementary Table 11……………………………………………………………..15

Supplementary Table 12……………………………………………………………..17

Supplementary Table 13……………………………………………………………..18

Supplementary Table 1: Results of group differences in functional connectivity between ET patients and HC for left M1 seed (Z threshold > 3.3, cluster p significance < 0.007). Coordinates are expressed in MNI standard space.

AAL anatomical area		Activated (mm^3)	Z max	MNI Coordinates x(mm)	y(mm)	z(mm)
**HC vs ET patients**						
Frontal lobes						
2	Precentral_R	4480	8.9	26	-30	66
3	Frontal_Sup_L	2984	10.5	-32	-8	66
4	Frontal_Sup_R	1944	7.3	22	-6	68
7	Frontal_Mid_L	1480	8.1	-30	-6	64
8	Frontal_Mid_R	472	5.1	44	0	52
19	Supp_Motor_Area_L	5472	7.6	0	-10	56
20	Supp_Motor_Area_R	2992	6.8	2	-10	56
Limbic lobes						
33	Cingulum_Mid_L	3200	7.6	-6	-4	46
34	Cingulum_Mid_R	424	4.8	2	6	42
Parietal lobes						
57	Postcentral_L	11728	17.9	-42	-28	66
58	Postcentral_R	7696	9.9	34	-34	72
59	Parietal_Sup_L	5616	9.5	-40	-42	56
60	Parietal_Sup_R	2344	7.0	34	-50	64
61	Parietal_Inf_L	7952	11.9	-56	-32	52
62	Parietal_Inf_R	280	5.1	32	-40	50
63	SupraMarginal_L	3856	10.6	-64	-28	38
64	SupraMarginal_R	256	5.6	62	-22	48
67	Precuneus_L	1696	5.9	-16	-56	60
68	Precuneus_R	1536	5.6	12	-44	66
69	Paracentral_Lobule_L	2040	6.2	-18	-12	64
70	Paracentral_Lobule_R	2168	6.1	6	-38	72
Cerebellum						
98	Cerebelum_4_5_R	48	3.9	14	-56	-18
100	Cerebelum_6_R	24	3.7	16	-58	-18

Abbreviations: HC, Healthy controls; AAL, Anatomical Automated Labeling Atlas; MNI, Montreal Neurological Institute.

Supplementary Table 2: Results of group differences in functional connectivity between ET patients and HC for left S1 seed (Z threshold > 3.3, cluster p significance < 0.007). Coordinates are expressed in MNI standard space.

AAL anatomical area		Activated (mm^3)	Z max	MNI Coordinates		
				x(mm)	y(mm)	z(mm)
**ET patients vs HC**						
Frontal lobes						
1	Precentral_L	3216	7.6	-14	-4	68
2	Precentral_R	4888	10.7	38	-10	64
3	Frontal_Sup_L	1352	10.4	-16	-2	70
4	Frontal_Sup_R	1904	11.3	36	-6	66
7	Frontal_Mid_L	128	5.9	-46	12	46
8	Frontal_Mid_R	384	9.5	50	-6	52
17	Rolandic_Oper_L	288	6.6	-50	-10	14
18	Rolandic_Oper_R	768	5.7	62	-10	14
19	Supp_Motor_Area_L	2056	10.1	-12	2	70
20	Supp_Motor_Area_R	1432	9.7	12	-14	72
Limbic lobes						
33	Cingulum_Mid_L	856	6.8	-10	-38	48
34	Cingulum_Mid_R	944	6.0	12	-42	50
Parietal lobes						
58	Postcentral_R	9336	9.0	52	-30	58
59	Parietal_Sup_L	3312	15.0	-40	-46	60
60	Parietal_Sup_R	3304	10.4	32	-62	56
61	Parietal_Inf_L	5848	15.7	-42	-48	60
62	Parietal_Inf_R	2392	7.3	54	-40	52
63	SupraMarginal_L	904	7.5	-64	-30	30
64	SupraMarginal_R	1368	7.8	60	-24	44
65	Angular_L	320	7.4	-36	-72	52
66	Angular_R	128	6.2	28	-58	42
67	Precuneus_L	1056	7.2	-16	-40	68
68	Precuneus_R	2208	7.5	16	-48	52
69	Paracentral_Lobule_L	2144	9.0	-16	-28	74
70	Paracentral_Lobule_R	1848	6.8	14	-44	52
**HC vs ET patients**						
Cerebellum						
91	Cerebelum_Crus1_L	40	4.1	-42	-46	-30
92	Cerebelum_Crus1_R	72	5.2	42	-52	-28

93	Cerebelum_Crus2_L	24	3.6	-44	-60	-42
98	Cerebelum_4_5_R	32	4.2	22	-48	-28
99	Cerebelum_6_L	200	5.0	-38	-46	-28
100	Cerebelum_6_R	1064	6.1	34	-52	-28
104	Cerebelum_8_R	488	5.1	18	-62	-40

Abbreviations: HC, Healthy controls; AAL, Anatomical Automated Labeling Atlas; MNI, Montreal Neurological Institute.

Supplementary Table 3: Results of group differences in functional connectivity between ET patients and HC for left SMA seed (Z threshold > 3.3, cluster p significance < 0.007). Coordinates are expressed in MNI standard space.

AAL anatomical area		Activated (mm^3)	Z max	MNI Coordinates x(mm)	y(mm)	z(mm)
**ET patients vs HC**						
Frontal lobes						
3	Frontal_Sup_L	2152	7.0	-20	8	56
4	Frontal_Sup_R	2912	6.4	18	4	52
7	Frontal_Mid_L	4384	7.4	-38	6	36
8	Frontal_Mid_R	2048	6.8	28	16	54
11	Frontal_Inf_Oper_L	2176	5.9	-36	12	16
12	Frontal_Inf_Oper_R	1288	7.7	38	4	26
13	Frontal_Inf_Tri_L	560	5.1	-48	14	24
14	Frontal_Inf_Tri_R	56	3.9	42	12	24
20	Supp_Motor_Area_R	2504	8.8	8	24	46
23	Frontal_Sup_Medial_L	512	5.5	2	26	50
24	Frontal_Sup_Medial_R	888	8.1	8	24	44
Limbic lobes						
33	Cingulum_Mid_L	688	5.8	0	-10	34
34	Cingulum_Mid_R	2560	6.9	10	20	44
**HC vs ET patients**						
Frontal lobes						
1	Precentral_L	6240	6.8	-36	-12	46
2	Precentral_R	2960	7.2	58	2	44
Parietal lobes						
57	Postcentral_L	7648	8.3	-56	-10	44
58	Postcentral_R	8296	7.4	44	-32	46
59	Parietal_Sup_L	1384	6.0	-42	-42	56
60	Parietal_Sup_R	2120	6.2	22	-60	60
61	Parietal_Inf_L	7744	7.9	-56	-42	42
62	Parietal_Inf_R	1400	7.4	42	-34	46
63	SupraMarginal_L	6184	8.2	-54	-36	34
64	SupraMarginal_R	5416	7.5	66	-24	38

Abbreviations: HC, Healthy controls; AAL, Anatomical Automated Labeling Atlas; MNI, Montreal Neurological Institute.

Supplementary Table 4: Results of group differences in functional connectivity between ET patients and HC for left thalamus seed (Z threshold > 3.3, cluster p significance < 0.007). Coordinates are expressed in MNI standard space.

AAL anatomical area		Activated (mm^3)	Z max	MNI Coordinates		
				x(mm)	y(mm)	z(mm)
**ET patients vs HC**						
Cerebellum						
91	Cerebelum_Crus1_L	432	5.8	-32	-68	-28
99	Cerebelum_6_L	312	5.6	-32	-68	-26
100	Cerebelum_6_R	80	5.6	30	-52	-20
111	Vermis_4_5	8	4.8	4	-48	6

Abbreviations: HC, Healthy controls; AAL, Anatomical Automated Labeling Atlas; MNI, Montreal Neurological Institute.

Supplementary Table 5: Results of group differences in functional connectivity between ET patients and HC for right cerebellar lobule IV-V seed (Z threshold > 3.3, cluster p significance < 0.007). Coordinates are expressed in MNI standard space.

AAL anatomical area		Activated (mm^3)	Z max	MNI Coordinates x(mm)	y(mm)	z(mm)
**HC vs ET patients**						
Frontal lobes						
1	Precentral_L	1312	6.8	-24	-14	74
2	Precentral_R	1544	6.3	62	2	22
3	Frontal_Sup_L	184	6.2	-22	-10	74
17	Rolandic_Oper_L	96	5.6	-48	-12	20
18	Rolandic_Oper_R	1872	5.9	58	4	16
19	Supp_Motor_Area_L	72	5.0	-12	-6	76
20	Supp_Motor_Area_R	48	3.8	12	-4	74
Cerebellum						
91	Cerebelum_Crus1_L	2840	7.5	-20	-92	-22
92	Cerebelum_Crus1_R	3144	7.1	26	-84	-18
93	Cerebelum_Crus2_L	544	6.2	-42	-62	-48
94	Cerebelum_Crus2_R	432	5.6	36	-36	-40
97	Cerebelum_4_5_L	160	5.2	-30	-34	-30
99	Cerebelum_6_L	808	5.4	-28	-64	-28
100	Cerebelum_6_R	3656	7.5	38	-34	-30
101	Cerebelum_7b_L	664	5.8	-28	-38	-42
102	Cerebelum_7b_R	376	4.9	18	-78	-54
103	Cerebelum_8_L	3664	6.2	-34	-46	-58
104	Cerebelum_8_R	5032	6.6	30	-50	-44
105	Cerebelum_9_L	40	4.1	-22	-48	-48
106	Cerebelum_9_R	96	4.8	20	-50	-44
107	Cerebelum_10_L	32	4.1	-28	-36	-40
108	Cerebelum_10_R	56	5.2	34	-38	-42

Abbreviations: HC, Healthy controls; AAL, Anatomical Automated Labeling Atlas; MNI, Montreal Neurological Institute.

Supplementary Table 6: Results of group differences in functional connectivity between ET patients and HC for right cerebellar lobule VI seed (Z threshold > 3.3, cluster p significance < 0.007). Coordinates are expressed in MNI standard space.

AAL anatomical area		Activated (mm^3)	Z max	MNI Coordinates x(mm)	y(mm)	z(mm)
**HC vs ET patients**						
Frontal lobes						
1	Precentral_L	960	8.8	-30	-20	74
3	Frontal_Sup_L	784	7	-20	-10	76
Cerebellum						
91	Cerebelum_Crus1_L	384	7	-46	-48	-30
92	Cerebelum_Crus1_R	3624	10.2	46	-42	-30
94	Cerebelum_Crus2_R	2488	9.4	36	-80	-46
98	Cerebelum_4_5_R	416	7.1	34	-32	-30
102	Cerebelum_7b_R	976	7.5	26	-78	-48
104	Cerebelum_8_R	3264	6.2	34	-68	-50
106	Cerebelum_9_R	240	6.2	20	-48	-44
108	Cerebelum_10_R	496	6.3	24	-36	-44

Abbreviations: HC, Healthy controls; AAL, Anatomical Automated Labeling Atlas; MNI, Montreal Neurological Institute.

Supplementary Table 7: Results of group differences in functional connectivity between ET patients and HC for right cerebellar lobule VIII seed (Z threshold > 3.3, cluster p significance < 0.007). Coordinates are expressed in MNI standard space.

AAL anatomical area		Activated (mm^3)	Z max	MNI Coordinates x(mm)	y(mm)	z(mm)
**HC vs ET patients**						
Cerebellum						
91	Cerebelum_Crus1_L	112	3.9	-10	-72	-28
92	Cerebelum_Crus1_R	816	4.9	10	-92	-26
93	Cerebelum_Crus2_L	784	5.7	2	-86	-30
94	Cerebelum_Crus2_R	3632	7.3	36	-80	-46
100	Cerebelum_6_R	2280	6.7	36	-48	-28
102	Cerebelum_7b_R	584	5.7	24	-74	-44

Abbreviations: HC, Healthy controls; AAL, Anatomical Automated Labeling Atlas; MNI, Montreal Neurological Institute.

Supplementary Table 8: Relationship of functional connectivity strength of M1 seed and clinical variables in ET patients (Z threshold > 3.3, cluster p significance < 0.007). Coordinates are expressed in MNI standard space.

a. Results of relationship with TRS

AAL anatomical area		Activated (mm^3)	Z max	MNI Coordinates x(mm)	y(mm)	z(mm)
**Positive relationship**						
Parietal lobes						
57	Postcentral_L	809	7.8	66	44	65
58	Postcentral_R	842	5.8	25	48	67
59	Parietal_Sup_L	230	5.4	56	30	57
61	Parietal_Inf_L	1611	9.8	68	43	59
63	SupraMarginal_L	837	9.2	73	48	53
64	SupraMarginal_R	173	7.6	24	45	56
66	Angular_R	257	6.5	28	33	60
68	Precuenus_R	272	7.4	37	32	59
**Negative relationship**						
Frontal lobes						
4	Frontal Sup R	461	4.0	30	62	18
5	Frontal_Sup_Orb_L	171	6.8	-26	62	-6
6	Frontal_Sup_Orb_R	149	7.0	28	60	-4
7	Frontal Mid L	269	6.0	-34	54	4
8	Frontal_Mid_R	1189	5.9	42	54	2
10	Front Mid Orb R	497	6.3	38	48	-10
12	Front Inf Oper R	299	5.1	40	2	26
13	Frontal Inf Tri L	120	4.3	-40	32	24
14	Fronta Inf Tri R	301	4.5	36	20	28
16	Frontal Inf Orb R	173	3.6	32	30	-16
19	Supp_Motor_Area_L	85	5.7	-4	-10	76
20	Supp_Motor_Area_R	326	6.7	10	-8	72
78	Thalamus_R	280	6.9	8	-18	10
Cerebellum						
91	Cerebellumm Crus 1 L	333	5.3	-34	-74	-28
92	Cerebellum Crus 1 R	146	5.0	34	64	-34
93	Cerebellumm Crus 2 L	167	4.6	-38	-76	-40
99	Cerebelum_6_L	149	6.0	-30	-66	-26
100	Cerebellum 6 R	110	5.3	32	-66	-28

b. Results of relationship with disease duration

AAL anatomical area		Activated (mm³)	Z max	MNI Coordinates x(mm)	y(mm)	z(mm)
**Negative relationship**						
Frontal lobes						
3	Frontal_Sup_L	110	5.1	-14	-10	70
4	Frontal_Sup_R	393	6.0	20	6	58
5	Frontal_Sup_Orb_L	154	5.9	-26	60	-6
6	Frontal_Sup_Orb_R	86	4.6	28	62	-4
7	Frontal_Mid_L	113	5.3	-40	46	8
8	Frontal_Mid_R	636	5.4	34	48	32
10	Frontal_Mid_Orb_R	239	5.1	36	50	-12
14	Frontal_Inf_Tri_R	124	4.2	46	32	6
20	Supp_Motor_Area_R	183	3.7	10	-4	64

Abbreviations: TRS, Fahn-Tolosa-Marin rating scale; AAL, Anatomical Automated Labeling Atlas; MNI, Montreal Neurological Institute.

Supplementary Table 9: Relationship of functional connectivity strength of thalamus seed and clinical variables in ET patients (Z threshold > 3.3, cluster p significance < 0.007). Coordinates are expressed in MNI standard space.

a. Results of relationship with TRS

AAL anatomical area		Activated (mm^3)	Z max	MNI Coordinates		
				x(mm)	y(mm)	z(mm)
**Positive relationship**						
Cerebellum						
91	Cerebelum_Crus1_L	804	6.6	-24	-88	-30
92	Cerebelum_Crus1_R	1169	7.9	24	-86	-20
93	Cerebelum_Crus2_L	742	7.3	-6	-84	-30
94	Cerebelum_Crus2_R	876	7.1	24	-90	-32
99	Cerebelum_6_L	499	8.0	-6	-62	-20
100	Cerebelum_6_R	329	5.6	12	-82	-18
113	Vermis_7	149	6.5	4	-78	-26
115	Vermis_9	20	4.9	4	-56	-30
**Negative relationship**						
Frontal lobes						
3	Frontal_Sup_L	884	9.0	-18	30	58
11	Frontal_Inf_Oper_L	173	5.0	-62	6	10
12	Frontal_Inf_Oper_R	279	3.4	40	8	14
18	Rolandic_Oper_R	816	6.0	44	-18	22
23	Frontal_Sup_Medial_L	394	6.8	0	40	36
24	Frontal_Sup_Medial_R	117	9.3	2	32	58
Parietal lobes						
57	Postcentral_L	601	7.9	-64	-12	16
58	Postcentral_R	1287	9.1	62	-10	20
64	SupraMarginal_R	501	5.4	48	-16	28

b. Results of relationship with disease duration

AAL anatomical area		Activated (mm^3)	Z max	MNI Coordinates		
				x(mm)	y(mm)	z(mm)
**Positive relationship**						

Cerebellum						
91	Cerebelum_Crus1_L	648	6.6	-38	-74	-26
92	Cerebelum_Crus1_R	735	7.2	48	-70	-38
93	Cerebelum_Crus2_L	775	9.1	-8	-86	-30
94	Cerebelum_Crus2_R	750	7.8	22	-92	-32
99	Cerebelum_6_L	215	7.2	-6	-80	-16
112	Vermis_6	167	6.5	-2	-64	-22

Abbreviations: TRS, Fahn-Tolosa-Marin rating scale; AAL, Anatomical Automated Labeling Atlas; MNI, Montreal Neurological Institute.

Supplementary Table 10: Relationship of functional connectivity strength of SMA seed and clinical variables in ET patients (Z threshold > 3.3, cluster p significance < 0.007). Coordinates are expressed in MNI standard space.

a. Results of relationship with TRS

AAL anatomical area		Activated (mm^3)	Z max	MNI Coordinates x(mm)	y(mm)	z(mm)
**Negative relationship**						
Frontal lobes						
1	Precentral_L	143	7.4	-26	-26	68

b. Results of relationship with disease duration

AAL anatomical area		Activated (mm^3)	Z max	MNI Coordinates x(mm)	y(mm)	z(mm)
**Negative relationship**						
Frontal lobes						
1	Precentral_L	711	7.2	-52	0	26
2	Precentral_R	185	5.0	48	6	40

Abbreviations: TRS, Fahn-Tolosa-Marin rating scale; AAL, Anatomical Automated Labeling Atlas; MNI, Montreal Neurological Institute.

Supplementary Table 11: Relationship of functional connectivity strength of cerebellar lobule IV-V seed and clinical variables in ET patients (Z threshold > 3.3, cluster p significance < 0.007). Coordinates are expressed in MNI standard space.

a. Results of relationship with TRS

AAL anatomical area		Activated (mm^3)	Z max	MNI Coordinates x(mm)	y(mm)	z(mm)
**Negative relationship**						
Frontal lobes						
1	Precentral_L	439	6.1	-38	-6	56
3	Frontal_Sup_L	123	5.3	-14	10	70
4	Frontal_Sup_R	67	3.9	16	58	32
12	Frontal_Inf_Oper_R	185	4.9	54	12	8
20	Supp_Motor_Area_R	134	5.4	12	12	68
23	Frontal_Sup_Medial_L	250	4.3	-6	48	18
24	Frontal_Sup_Medial_R	134	4.5	6	64	28
Cerebellum						
91	Cerebelum_Crus1_L	558	6.5	-8	-72	-26
92	Cerebelum_Crus1_R	262	5.7	36	-68	-34
93	Cerebelum_Crus2_L	570	7.3	-40	-70	-50
99	Cerebelum_6_L	856	8.8	-16	-58	-20
100	Cerebelum_6_R	623	8.7	32	-42	-38
101	Cerebelum_7b_L	247	8.6	-42	-50	-46
103	Cerebelum_8_L	1033	6.8	-26	-66	-44
104	Cerebelum_8_R	827	7.0	18	-66	-42
105	Cerebelum_9_L	593	8.8	-8	-50	-46
106	Cerebelum_9_R	434	8.8	12	-42	-56
107	Cerebelum_10_L	85	7.6	-26	-42	-42

b. Results of relationship with disease duration

AAL anatomical area		Activated (mm^3)	Z max	MNI Coordinates x(mm)	y(mm)	z(mm)
**Negative relationship**						
Frontal lobes						
1	Precentral_L	184	5.7	-50	0	36

Cerebellum						
91	Cerebelum_Crus1_L	172	6.1	-42	-62	-38
96	Cerebelum_3_R	106	7.0	18	-34	-28
97	Cerebelum_4_5_L	380	7.7	-14	-52	-12
99	Cerebelum_6_L	435	8.0	-16	-58	-18
100	Cerebelum_6_R	217	6.8	28	-62	-22
103	Cerebelum_8_L	534	7.2	-14	-58	-58
105	Cerebelum_9_L	327	7.2	-14	-48	-52
107	Cerebelum_10_L	32	5.8	-18	-32	-44
111	Vermis_4_5	79	6.1	-2	-54	0
114	Vermis_8	69	6.2	2	-72	-38

Abbreviations: TRS, Fahn-Tolosa-Marin rating scale; AAL, Anatomical Automated Labeling Atlas; MNI, Montreal Neurological Institute.

Supplementary Table 12: Relationship of functional connectivity strength of cerebellar lobule VI seed and clinical variables in ET patients (Z threshold > 3.3, cluster p significance < 0.007). Coordinates are expressed in MNI standard space.

a. Results of relationship with TRS

AAL anatomical area		Activated (mm^3)	Z max	MNI Coordinates x(mm)	y(mm)	z(mm)
Frontal lobes						
1	Precentral_L	260	5.1	-36	-6	62
11	Frontal_Inf_Oper_L	171	6.1	-48	14	4
23	Frontal_Sup_Medial_L	356	5.4	2	50	46
Cerebellum						
91	Cerebelum_Crus1_L	302	6.4	-40	-52	-34
97	Cerebelum_4_5_L	578	7.3	-4	-56	-6
98	Cerebelum_4_5_R	128	5.0	16	-52	-18
99	Cerebelum_6_L	220	7.0	-30	-40	-36
103	Cerebelum_8_L	427	5.4	-24	-66	-48
105	Cerebelum_9_L	301	7.3	-10	-42	-44
106	Cerebelum_9_R	153	4.6	16	-50	-46
111	Vermis_4_5	345	5.0	2	-52	-20

b. Results of relationship with disease duration

AAL anatomical area		Activated (mm^3)	Z max	MNI Coordinates x(mm)	y(mm)	z(mm)
**Negative relationship**						
Cerebellum						
91	Cerebelum_Crus1_L	339	6.6	-48	-64	-30
92	Cerebelum_Crus1_R	139	6.7	52	-56	-36
94	Cerebelum_Crus2_R	31	5.0	44	-44	-40
97	Cerebelum_4_5_L	302	5.7	-22	-28	-30
99	Cerebelum_6_L	261	6.7	-34	-44	-36
103	Cerebelum_8_L	457	6.0	-10	-62	-48

Abbreviations: TRS, Fahn-Tolosa-Marin rating scale; AAL, Anatomical Automated Labeling Atlas; MNI, Montreal Neurological Institute.

Supplementary Table 13: Relationship of functional connectivity strength of cerebellar lobule VIII seed and clinical variables in ET patients (Z threshold > 3.3, cluster p significance < 0.007). Coordinates are expressed in MNI standard space.

a. Results of relationship with TRS

AAL anatomical area		Activated (mm^3)	Z max	MNI Coordinates x(mm)	y(mm)	z(mm)
**Negative relationship**						
Frontal lobes						
1	Precentral_L	22	4.2	-56	2	40
5	Frontal_Sup_Orb_L	33	4.8	-18	22	-14
15	Frontal_Inf_Orb_L	157	6.3	-28	32	-12
Cerebellum						
94	Cerebelum_Crus2_R	91	9.1	46	-66	-42
100	Cerebelum_6_R	91	6.3	36	-42	-38

b. Results of relationship with disease duration

AAL anatomical area		Activated (mm^3)	Z max	MNI Coordinates x(mm)	y(mm)	z(mm)
**Negative relationship**						
Cerebellum						
91	Cerebelum_Crus1_L	271	6.4	-42	-62	-38
92	Cerebelum_Crus1_R	394	7.1	38	-66	-30
93	Cerebelum_Crus2_L	199	5.2	-36	-68	-38
94	Cerebelum_Crus2_R	229	8.0	38	-44	-42
98	Cerebelum_4_5_R	108	4.3	20	-48	-18
100	Cerebelum_6_R	345	5.5	18	-60	-16
101	Cerebelum_7b_L	152	7.6	-14	-72	-44
102	Cerebelum_7b_R	129	6.1	40	-56	-48
103	Cerebelum_8_L	684	6.8	-22	-54	-48
106	Cerebelum_9_R	236	5.4	14	-48	-54

Abbreviations: TRS, Fahn-Tolosa-Marin rating scale; AAL, Anatomical Automated Labeling Atlas; MNI, Montreal Neurological Institute.
